# Missing Iron-Oxidizing Acidophiles Highly Sensitive to Organic Compounds

**DOI:** 10.1264/jsme2.ME16086

**Published:** 2016-06-29

**Authors:** Nagayoshi Ueoka, Atsushi Kouzuma, Kazuya Watanabe

**Affiliations:** 1 School of Life Sciences, Tokyo University of Pharmacy and Life Sciences1432–1 Horinouchi, Hachioji 192–0392, TokyoJapan

**Keywords:** lithotroph, *Acidithiobacillus ferrooxidans*, 16S rRNA gene, HiPIP, rusticyanin

## Abstract

The genus *Acidithiobacillus* includes iron-oxidizing lithoautotrophs that thrive in acidic mine environments. *Acidithiobacillus ferrooxidans* is a representative species and has been extensively studied for its application to the bioleaching of precious metals. In our attempts to cultivate the type strain of *A. ferrooxidans* (ATCC 23270^T^), repeated transfers to fresh inorganic media resulted in the emergence of cultures with improved growth traits. Strains were isolated from the resultant culture by forming colonies on inorganic silica-gel plates. A representative isolate (strain NU-1) was unable to form colonies on agarose plates and was more sensitive to organics, such as glucose, than the type strain of *A. ferrooxidans*. Strain NU-1 exhibited superior growth traits in inorganic iron media to those of other iron-oxidizing acidithiobacilli, suggesting its potential for industrial applications. A draft genome of NU-1 uncovered unique features in catabolic enzymes, indicating that this strain is not a mutant of the *A. ferrooxidans* type strain. Our results indicate that the use of inorganic silica-gel plates facilitates the isolation of as-yet-unexamined iron-oxidizing acidithiobacilli from environmental samples and enrichment cultures.

The genus *Acidithiobacillus* includes lithoautotrophic acidophiles that conserve energy for growth by the oxidation of inorganic compounds, such as ferrous iron, reduced sulfur compounds, and hydrogen, under aerobic conditions (11). *Acidithiobacillus ferrooxidans* is a representative iron-oxidizing acidophile that has been extensively studied for its application to the bioleaching of precious metals (26). Bioleaching is employed to extract metals from their ores through the use of living organisms, such as iron- and sulfur-oxidizing bacteria, and is widely used for metal recovery from low-grade ores and mineral concentrates (2). Physiological and genomic studies have identified iron-oxidizing pathways in *A. ferrooxidans*, which have contributed to a better understanding of how iron-oxidizing acidophiles conserve energy for growth (20, 26).

Many iron-oxidizing acidithiobacilli have been isolated from the environment, and their diversity and taxonomy have been studied (1). It has been suggested that the genus *Acidithiobacillus* is comprised of multiple species with different ferrous iron oxidation pathways (1). In addition to *A. ferrooxidans*, three new species, *A. ferrivorans* (7), *A. ferridurans* (8), and *A. ferriphilus* (5), have recently been proposed. Although the four species in the genus *Acidithiobacillus* are phylogenetically closely related to each other, these studies have suggested that acidithiobacilli in the environment are more diverse than previously considered.

A recent study engineered *A. ferrooxidans* for the production of isobutyrate from carbon dioxide (13). This indicates the potential of acidithiobacilli for the production of value-added chemicals, whereas productivity is lower than that required for commercial production primarily due to their low growth capacities. On the other hand, the growth of acidithiobacilli is known to be promoted in the presence of a cathodic electrode that regenerates ferrous iron from ferric iron (16). However, iron-oxidizing acidophiles with high catabolic abilities are still desired for industrial applications, in particular the bioleaching of precious metals.

We have investigated *A. ferrooxidans* in order to elucidate the molecular mechanisms underlying the electrode-promoted growth of acidithiobacilli. In the course of our research, we successfully isolated iron-oxidizing acidophiles represented by strain NU-1 from a subculture prepared to maintain *A. ferrooxidans* strain ATCC 23270^T^ by forming colonies on inorganic silica-gel plates (4, 10, 22). We herein report the physiological and genomic characteristics of this isolate and suggest that this is a novel strain of acidithiobacilli that has remained unknown behind the wide use of the type strain due to its high sensitivity to organic compounds.

## Materials and Methods

### Bacterial strains used in this study

The bacterial strains used in the present study were *A. ferrooxidans* ATCC 23270^T^, *A. ferrooxidans* DSM 14882^T^, *A. ferrivorans* DSM 22755^T^, *A. ferridurans* JCM 18981^T^, and *A. ferriphilus* JCM 7812. These strains were obtained from the respective culture-collection centers.

### Culture conditions and characterizations

The strains were grown in Fe9K medium (23) at 30°C and maintained by being transferred to fresh liquid medium every two weeks. Fe9K medium was prepared by adding FeSO_4_·7H_2_O (30 g L^−1^) to 9K medium that contained (L^−1^) 4.25 g (NH_4_)_2_SO_4_, 0.14 g KCl, 0.07 g K_2_HPO_4_, 0.7 g MgSO_4_·7H_2_O, and 0.02 g Ca(NO_3_)_2_·4H_2_O, and pH was adjusted using 10 N H_2_SO_4_ solution. Routine liquid cultivation was conducted in 300-mL baffle flasks containing 100 mL of Fe9K medium at pH 2.0. Medium was solidified either with agarose (1.5%; LO3, Takara Bio, Shiga, Japan) or colloidal silica (Snowtex-OXS, Nissan Chemical Industries, Tokyo, Japan). In order to prepare Fe9K silica-gel plates, a colloidal silica suspension was mixed with five-fold concentrated 9K medium at a ratio of 1:4, and FeSO_4_·7H_2_O was slowly added to the mixture at a final concentration of 100 mM while gently stirring the solution. After pH was adjusted to 2.0, the solution was poured into glass petri dishes, and autoclaved for 20 min for sterilization and solidification. Solidified colloidal silica was gently dried and used as a silica-gel plate. During the cultivation of acidithiobacilli on silica-gel plates, petri dishes were laid upside down in an incubator at 30°C, and a drop of water was added to the inside of the dish cap in order to maintain humidity.

Growth in a liquid culture was checked by counting cells in the culture using a fluorescence microscope (BX60, Olympus) after cells were completely dispersed and stained with 4′,6-diamidino-2-phenylindole (DAPI). Sensitivity to organic compounds was examined in Fe9K medium (pH 2.0) supplemented with glucose (2.5 mM, 5 mM, 10 mM, or 20 mM) or glycerol (2.5 mM, 5 mM, or 10 mM). In growth examinations, three independent cultures were analyzed for each condition, and data were statistically analyzed by the *t*-test or Tukey’s range test.

### Genomic analyses

Cells were grown in 100 mL of Fe9K medium (pH 2.0) and harvested by centrifugation at 12,000×*g* for 20 min. Cells were washed with medium and subjected to DNA extraction using a Favorprep tissue genomic DNA extraction mini kit (Favorgen Biotech, Ping-Tung, Taiwan). Extracted DNA was treated with RNase (Nippon Gene, Toyama, Japan), and further purified using a NucleoSpin gDNA clean-up kit (Macherey-Nagel, Düren, Germany). Genome sequencing was conducted using an Illumina Miseq sequencer, and reads were assembled to reconstruct contigs using CLC genomics workbench version 6.5.1 (CLC Bio Japan, Tokyo, Japan). Coding sequences (CDSs) were predicted by MetaGeneAnnotator (19). Annotation of the predicted CDSs was performed by a conserved-domain search (15) against the COG database in the NCBI database with an *E*-value threshold of 0.01 (19). *In silico* DNA-DNA hybridization (DDH) between the draft genome of strain NU-1 and previously reported genomes of *A. ferrooxidans* and *A. ferrivorans* was conducted using GGDC2 (17). The nucleotide sequences determined in the present study have been deposited into the DDBJ Sequence Read Archive database under accession number DRA004250.

### Phylogenetic analyses

Cells were selected from a colony, grown in Fe9K medium, harvested by centrifugation, and washed with fresh 9K medium and TE buffer. DNA was extracted from cells using DNAzol (Invitrogen, California, USA) and used for the PCR amplification and sequencing of a partial fragment of the 16S rRNA gene. PCR amplification was performed using the primers Bac331f (TCCTACGGGAGGCAGCAGT) and Bac797r (GGACTACCAGGGTCTAATCCTGTT) according to methods described elsewhere (18). In addition, genes for 16S rRNA, high-potential iron-sulfur protein (HiPIP), and rusticyanin were retrieved from the draft genome of strain NU-1. Phylogenetic analyses were conducted together with reference sequences obtained from the NCBI database (http://www.ncbi.nlm.nih.gov/). The alignment of sequences and construction of neighbor-joining trees were conducted using the MEGA program (24). Nucleotide sequences for the 16S rRNA, rusticyanin, and HiPIP genes in strain NU-1 have been deposited in the DDBJ, EMBL, and NCBI nucleotide sequence databases under accession no. LC115032, LC115033, and LC115034, respectively.

## Results and Discussion

### Isolation of strain NU-1

**Fig. 1:**
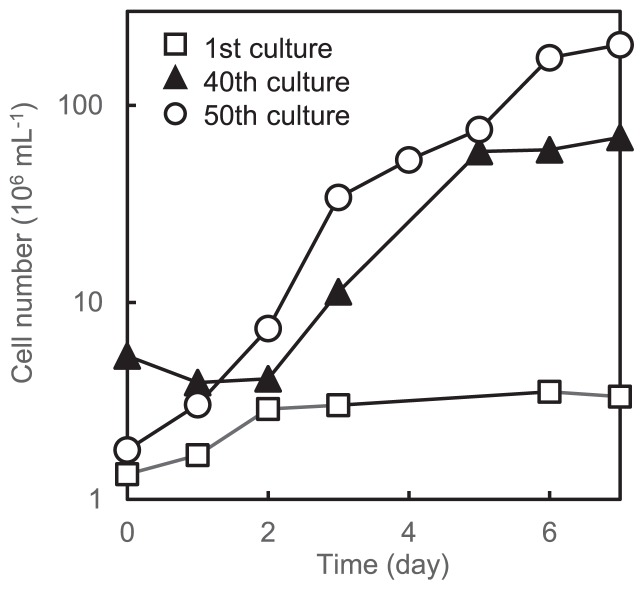
Growth curves of subcultures originated from *A. ferrooxidans* ATCC 23270^T^, showing the acceleration of growth during repeated transfers. Cell numbers were determined by the DAPI count.

*A. ferrooxidans* strain ATCC 23270^T^ was obtained from the ATCC culture collection and grown in Fe9K medium. We found that repeated transfers to fresh medium resulted in the emergence of cultures with improved growth traits (Fig. 1). After 50 repeated transfers, the specific growth rate and final cell density of the culture were 143-fold and 50-fold higher, respectively, than those for the initial-stage cultures (within several transfers). Two possible reasons have been considered for improved growth: the mutants of strain ATCC 23270^T^ with high growth capacities arose during repeated cultivation, and the culture was contaminated with small numbers of other microbes that gradually increased during repeated cultivation in terms of their high growth capacities.

**Fig. 2:**
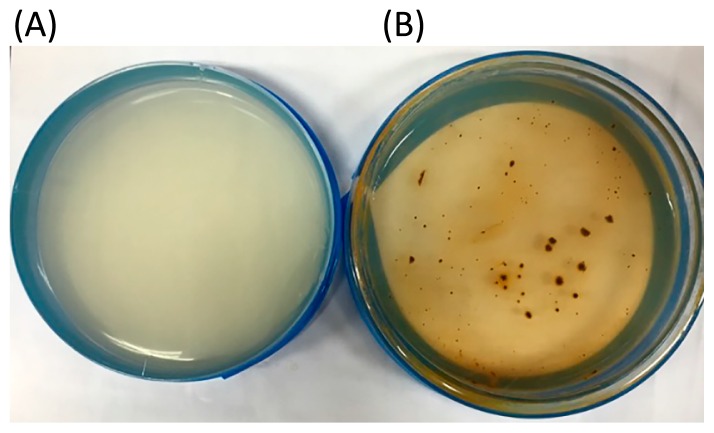
Appearance of agarose (A) and silica-gel (B) plates 10 day after streaking with a liquid culture of strain NU-1.

In order to examine these possibilities, we attempted to isolate microbes present in the resultant culture (the 50th culture). Previous studies have used agarose plates for the colony isolation and purification of acidithiobacilli, including strain ATCC 23270^T^ and its derivatives (7, 13). However, when serial dilutions of the resultant culture were streaked on Fe9K agarose plates, no colony formed on them. Therefore, we examined other methods for colony isolation and found that the use of silica-gel plates facilitated colony formation from the resultant culture (Fig. 2). Silica-gel plates have been used in a study to isolate iron-oxidizing *Leptospirillum* from environmental samples (22). However, these plates have not been used to isolate acidithiobacilli, and researchers consider silica gel to be a less convenient material than agar and agarose (9). In the present study, we optimized methods to prepare and use silica-gel plates containing Fe9K medium and successfully applied them to the isolation and maintenance of strain NU-1. In Fig. 2B, the brownish colors of colonies indicate that ferrous iron in the medium was oxidized to ferric iron. Colonies were selected from Fe9K silica-gel plates and purified by repeated transfer between liquid media and silica-gel plates.

**Fig. 3:**
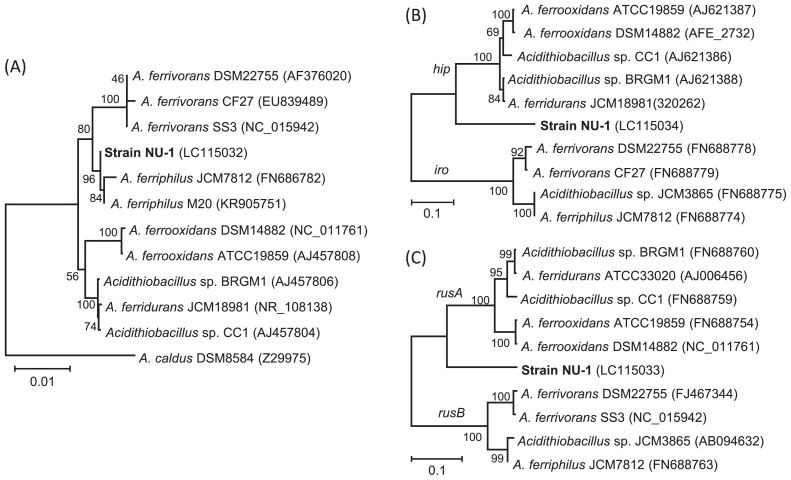
Neighbor-joining trees based on 16S rRNA (A), HiPIP (B), and rusticyanin (C) gene sequences showing phylogenetic relationships between strain NU-1 and known iron-oxidizing acidithiobacilli. Accession numbers are indicated in parentheses. Numbers at branch nodes are bootstrap values (100 trials); only values greater than 50 are shown.

Fragments of 16S rRNA genes were PCR-amplified from 3 isolates formed on Fe9K silica-gel plates, and their sequences were investigated. We found that their 16S rRNA gene sequences were 100% identical to each other (data not shown), whereas these sequences were different from that of *A. ferrooxidans*. Phylogenetic analyses showed that they were more closely related to *A. ferriphilus* (5) than *A. ferrooxidans* (refer to Fig. 3A for its phylogenetic position). This result prompted us to further analyze the isolates in order to assess whether they represent a novel type of acidithiobacilli. Since these isolates are phylogenetically identical, a representative strain (NU-1) was used in subsequent examinations.

### Characteristics of strain NU-1

In the following examinations, strain DSM 14882^T^ was used as the representative of *A. ferrooxidans*. This was because, in contrast to ATCC 23270^T^, strain DSM 14882^T^ constantly formed colonies on agarose plates after repeated subculturing, and the nucleotide sequences of genes, including the 16S rRNA gene, for DSM 14882^T^ are 100% identical to those stored in the databases for the type strain of *A. ferrooxidans*.

**Fig. 4:**
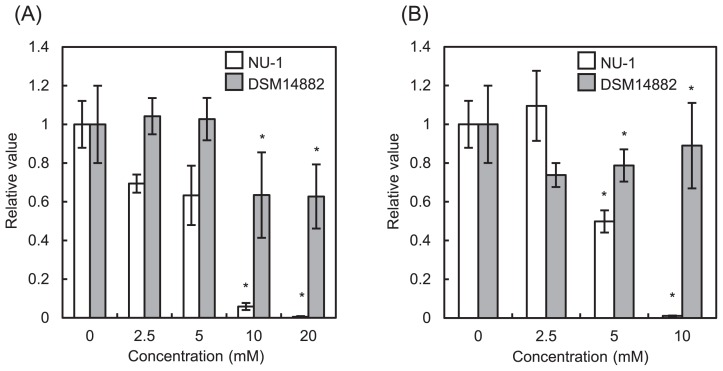
Effects of organic compounds on the growth of strains NU-1 and DSM 14882^T^. Specific growth rates relative to those without an organic compound are shown. Glucose (A) or glycerol (B) was added at a given concentration. Data and error bars represent means and SDs, respectively (*n*=3). Asterisks indicate significant differences between values for strains NU-1 and DSM 14882^T^ (*P* <0.05, *t*-test).

In contrast to the other acidithiobacilli obtained from the culture collections, strain NU-1 was unable to form colonies on agarose plates (Fig. 2). We attributed this to strain NU-1 being more sensitive to organic compounds than the other acidithiobacilli and examined its growth in the presence of different concentrations of glucose and glycerol (Fig. 4). The growth of strain NU-1 diminished at relatively low concentrations of glucose and glycerol, confirming that NU-1 is more sensitive to organics than *A. ferrooxidans*. A previous study reported that iron-oxidizing acidithiobacilli are sensitive to organics and unable to form colonies on agar plates (25). However, we found that acidithiobacilli obtained from culture collections were able to form colonies when molecular biology-grade agarose was used to solidify culture medium. Based on these results, we suggest that strain NU-1 represents as-yet-unexamined acidithiobacilli that are highly sensitive to organics and unable to form colonies on molecular biology-grade agarose plates.

**Fig. 5:**
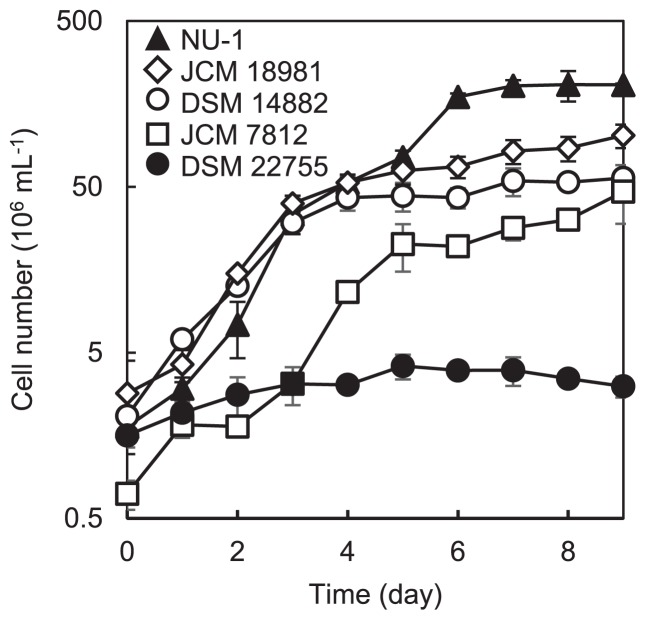
Growth characteristics of strain NU-1 in Fe9K medium. The growth of NU-1 in a shaken liquid culture was compared with those of representative iron-oxidizing acidithiobacilli, including *A. ferrooxidans* DSM 14882^T^, *A. ferrivorans* DSM 22755^T^, *A. ferridurans* JCM 18981^T^, and *A. ferriphilus* JCM 7812. Data points and error bars represent means and SDs, respectively (*n*=3).

The growth characteristics of strain NU-1 in Fe9K medium were compared to those of representative iron-oxidizing strains from the four species of the genus *Acidithiobacillus* (Fig. 5). We found that final cell concentrations in Fe9K liquid cultures of strain NU-1 were significantly higher than those of the other acidithiobacilli (as assessed by Tukey’s range test). Furthermore, the maximum specific growth rate of stain NU-1 was 8-fold higher than that of strain DSM 14882^T^. Since ferrous iron (added at the same concentration) was completely consumed in these cultures, strain NU-1 is considered to show a higher cell yield than those of the other acidithiobacilli. These results suggest that strain NU-1 is a potent acidophile and applicable to industrial processes. It currently remains unclear whether growth capacity in inorganic media is linked to sensitivity to organics, and it will be of interest to investigate overlaps in the molecular mechanisms underlying these physiological traits.

### Genomic and molecular features of strain NU-1

The assembly of a total of 34,672,023 paired-end reads from the MiSeq sequencer generated 69 contigs with an average length of 36,768 bp. The total length of the draft genome of strain NU-1 (comprised of these contigs) is 2,536,979 bp, in which 2,521 CDSs (including partial CDSs) are predicted. The genome encodes the Calvin-Benson-Bassham cycle for CO_2_ fixation, *rus* and *pet*I operons involved in iron oxidation, genes for sulfur metabolism, and those for nitrogen fixation. These are also coded in the two currently available genomes of acidithiobacilli (14, 26), suggesting that strain NU-1 harbors similar central metabolic pathways to those of the known iron-oxidizing acidithiobacilli. DDH values between the draft genome of strain NU-1 and the genomes of *A. ferrooxidans* (26) and *A. ferrivorans* (14) were 29% and 51%, respectively.

Strain NU-1 was phylogenetically characterized using the full-length sequences of genes for 16S rRNA, HiPIP, and rusticyanin retrieved from the draft genome (Fig. 3). These genes have been used to classify bacteria affiliated with the genus *Acidithiobacillus* (1). As described above, rRNA-gene analyses indicate that strain NU-1 is more closely related to *A. ferriphilus* (100% identical) than *A. ferrooxidans* (98.8%) (Fig. 3A); however, further examinations (*e.g.*, DDH) are needed in order to establish whether strain NU-1 is affiliated with *A. ferriphilus*.

On the other hand, genome analyses revealed the unique features of catabolic genes in strain NU-1. A previous study reported that HiPIP is widely distributed in acidithiobacilli and plays important roles in iron and sulfur oxidation (1). For example, HiPIP in *A. ferrooxidans* (referred to as Hip) is considered to be involved in sulfur oxidation (3), whereas that in other strains (Iro) may be the first electron acceptor from Fe(II) oxidation (6, 12). The present phylogenetic analysis suggests that HiPIP in strain NU-1 is a member of Hip, while it is distantly related to the known Hip proteins (Fig. 3B). In addition, we found that strain NU-1 has a unique rusticyanin (Fig. 3C). Rusticyanin, a blue copper protein involved in electron transfer in the iron-oxidizing pathway, has been classified into two types, RusA and RusB, which are considered to function differently in the pathway (1, 21). We found that NU-1 rusticyanin may be affiliated with RusA, while it is distantly related to known RusA rusticyanins. It will be of interest to examine how novel HiPIP and rusticyanin in strain NU-1 function in the iron-oxidizing pathway. Since the nucleotide sequences of genes in NU-1 are markedly different from those in the type strain of *A. ferrooxidans*, we conclude that NU-1 is not a mutant of *A. ferrooxidans* that emerged during subculturing in our laboratory.

## Conclusion

The present study isolated strain NU-1 from a subculture of *A. ferrooxidans* ATCC 23270^T^ using inorganic silica-gel plates. Physiological and genomic analyses indicate that strain NU-1 is an acidophile that is distinct from ATCC 23270^T^. Since we did not cultivate any acidophile other than ATCC 23270^T^ or treat acidic environmental samples at the time when we isolated strain NU-1, we consider NU-1 to exist as a minor population in the ATCC stock culture. Although this strain grows actively in inorganic Fe9K medium, it is unable to form colonies on agarose plates due to its high sensitivity to organic compounds. Highly organic-sensitive acidithiobacilli such as strain NU-1 may have escaped isolation and characterization despite their superior growth capacities in inorganic media. Therefore, our results suggest that *A. ferrooxidans* needs to be used cautiously after colony isolation on agarose plates. We also suggest that the use of inorganic silica-gel plates facilitates the isolation of as-yet-uncultivated acidithiobacilli from environmental samples. In future studies, we will identify how organic compounds inhibit the growth of strain NU-1. In these studies, comparative analyses of the draft genome of NU-1 and the genomes of other acidithiobacilli will be the primary step.
